# Supplementary material for: The interactome of intact mitochondria by cross-linking mass spectrometry provides evidence for coexisting respiratory supercomplexes
Source: Mol Cell Proteomics. 2017 Dec 8;17(2):216–32. doi: 10.1074/mcp.RA117.000470 (PMC5795388; doi:10.1074/mcp.RA117.000470)
Supplement: Supplemental Data [file supp_RA117.000470_133922_0_supp_23978_fzffwf.zip › spectra_annotation/mito_DR_spectra_annotation/265-1-4-1-6-1.pdf]

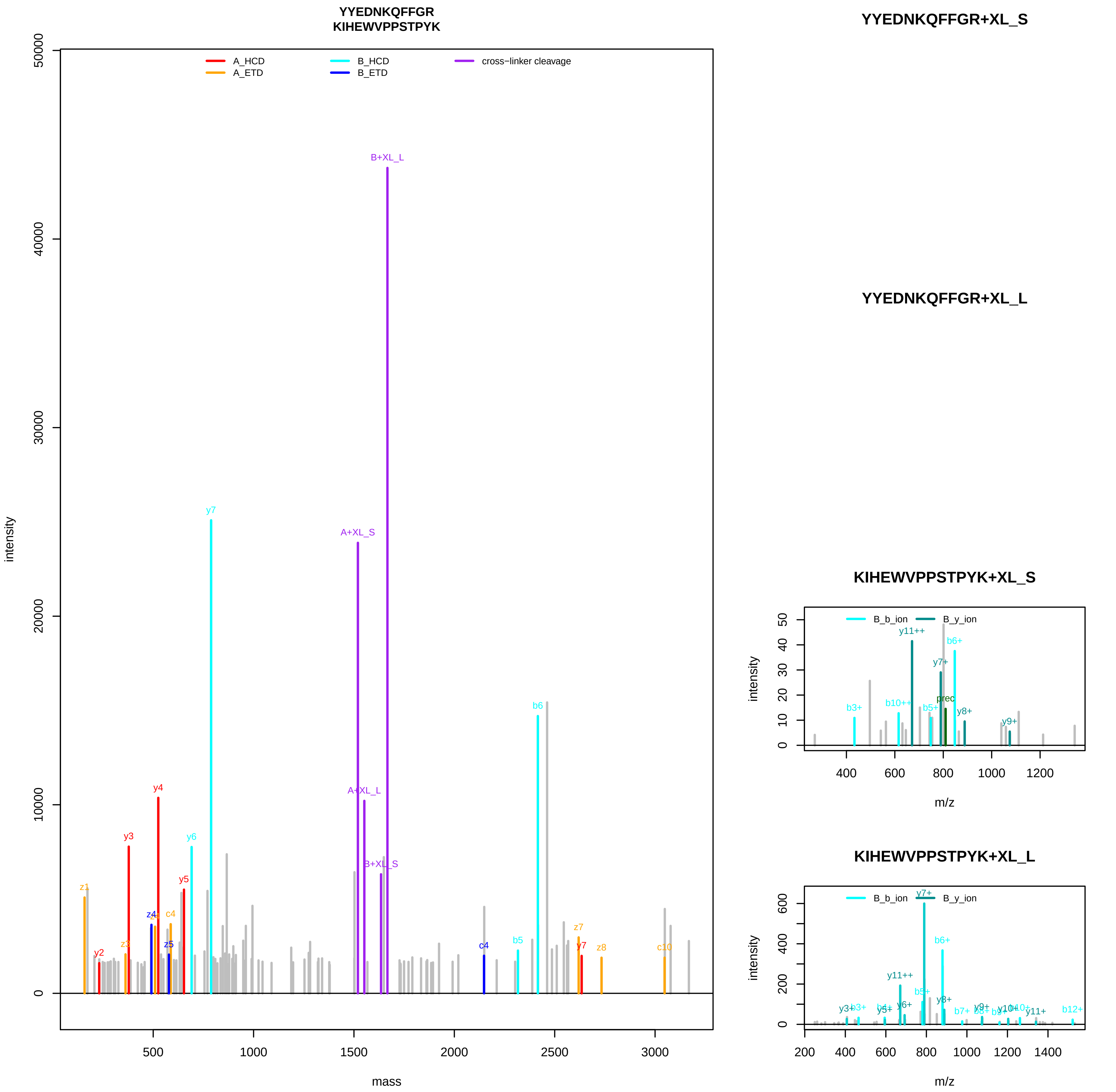
YYEDNKQFFGR
KIHEWVPPSTPYK
A_HCD
A_ETD
B_HCD
B_ETD
cross−linker cleavage
B+XL_L
A+XL_S
A+XL_L
B+XL_S
y7
y6
y5
y4
y3
y2
z1
z3
z4
z5
c4
b5
b6
z7
y7
z8
c10
intensity
mass
YYEDNKQFFGR+XL_S
YYEDNKQFFGR+XL_L
KIHEWVPPSTPYK+XL_S
B_b_ion
B_y_ion
y11++
b6+
y7+
prec
b10++
b5+
b3+
y8+
y9+
m/z
KIHEWVPPSTPYK+XL_L
y11++
b5+
y8+
y6+
y5+
y3+
b3+
b7+
y9+
y10+
y11+
b12+
